# Voices From the Field: Community‐Engaged Qualitative Study on Veterinary Service Delivery Gaps in Aari Zone, Southern Ethiopia

**DOI:** 10.1002/vms3.71173

**Published:** 2026-08-14

**Authors:** Yebelayhun Mulugeta, Taye Shibabaw, Haile Delelegn, Belete Jorga

**Affiliations:** ^1^ Department of Veterinary Medicine Jinka University Jinka Southern Ethiopia; ^2^ Department of Animal Science Jinka University Jinka Southern Ethiopia

**Keywords:** Aari Zone, livestock health, Southern Ethiopia, Qualitative Study, veterinary service gaps

## Abstract

Ethiopia has the largest livestock population in Africa, yet veterinary service delivery remains inadequate, particularly in remote rural and pastoral areas, undermining livestock health and rural livelihoods. Persistent gaps in workforce, infrastructure and supply chains exacerbate disease risks and economic vulnerability. Despite the recognized value of participatory approaches for improving service equity and sustainability, evidence from Southern Ethiopia remains limited. This study aimed to explore community experiences and perceptions of veterinary service delivery in the Aari Zone of Southern Ethiopia, identifying key barriers and opportunities for improvement through a community‐engaged qualitative approach. Six semi‐structured focus group discussions (FGDs) were conducted with 54 participants across four districts, and data were analysed using reflexive thematic analysis. Three major themes emerged: (1) Availability and Accessibility, such as severe shortage and instability of veterinary professionals, long travel distances (up to 4 h), inadequate clinics and cattle crushes, and lack of emergency/night‐time services; (2) Quality and Resources, including irregular drug and vaccine supply, absence of diagnostic facilities, low professional skill levels and poor infrastructure; and (3) Economic and Cultural Factors, consisting of high private drug costs, poverty and forage scarcity and continued reliance on traditional practices where modern services fail, despite no cultural resistance to modern care. This study provides the first qualitative evidence from Aari Zone situating local veterinary service gaps within Ethiopia's broader extension system. Findings underscore the need for integrated policy responses, including workforce stabilization, mobile veterinary units, diagnostic capacity building, gender‐sensitive training and affordability measures. Embedding veterinary services within farmer training centres and adopting One Health principles can enhance resilience and equity in rural animal health systems.

## Graphical abstract

This study explores veterinary service gaps in Aari Zone, Southern Ethiopia, using Community‐Engaged Qualitative Studyc. Key challenges include professional shortages, poor infrastructure, limited drug and diagnostic supplies and reliance on traditional practices. Findings highlight the need for systemic improvements in the veterinary workforce, infrastructure and supply chains to enhance livestock health and service accessibility.

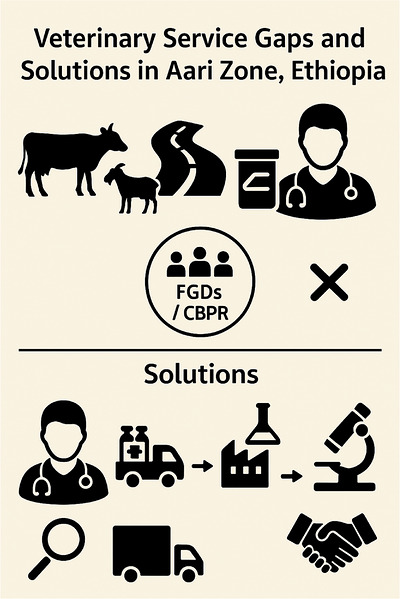

## 1 Introduction

Livestock production is an integral part of the agricultural system in Ethiopia. Ethiopia has the largest livestock population in Africa, possessing more than 70 million cattle, 95.4 million small ruminants (42.9 million sheep and 52.5 million goats), 8.1 million camels, 13.33 million equines and 57 million chickens. This livestock sector has contributed a considerable portion to the economy of the country and is still promising to rally around the economic development of the country. Livestock production remains crucial and represents a major asset among resource‐poor smallholder farmers by providing milk, meat, skin, manure and traction force (CSA 2021). In Ethiopia, livestock production contributes 40% of the agricultural gross domestic product (GDP) and 20% of the total GDP without considering other contributions like the provision of traction power, organic fertilizers and as a modes of transport (Aklilu et al. 2002). The contribution of livestock to the national economy, particularly foreign currency earnings, is through the exportation of live animals, meat, skin and hides (MoARD 2008).

Despite this, the economic contribution made by this enormous amount of cattle is not satisfactory. Numerous factors affect livestock production and productivity, of which livestock disease is one of the most important. This is mainly due to endemic and transboundary cattle diseases. Addressing problems related to the health of cattle in remote areas with a limited number of veterinarians and infrastructure, particularly during outbreaks, is another issue that seriously damages their potential economic contribution (Abel et al. 2018; CSA 2021).

Service delivery gaps are another bottleneck. Several studies in different parts of Ethiopia have identified different veterinary service gaps. Many rural and pastoral communities face poor access to veterinary services due to inadequate infrastructure, insufficient clinics and logistical challenges (Gizaw et al. 2021; Tessema and Negash 2022; Desta 2015; Jilo 2016; H. Kebede et al. 2014). There is a widespread lack of trained veterinary professionals, with many clinics relying on underqualified staff or community‐based animal health workers (CAHWs) who often lack standardized training and supervision (Nyokabi et al. 2024; Alafiatayo et al. 2022; Fedlu and Seid 2019; Larrateguy et al. 2019; Bessler et al. 2024). Both public and private sectors report frequent shortages of essential drugs, diagnostic materials and proper storage facilities, leading to compromised service quality (G. Kebede et al. 2025; Tafa et al. 2023; Tessema and Negash 2022; Jibat et al. 2015; Mekonen et al. 2024). Farmers often prefer public services for their affordability and trust, but turn to private providers for drug availability and timeliness, though both sectors have notable weaknesses (G. Kebede et al. 2025; Tafa et al. 2023; Mulugeta et al. 2019; Jibat et al. 2015; H. Kebede et al. 2014).

Veterinary service coverage, particularly in the South Omo Zone (including the Aari Zone) of Ethiopia, faces several challenges. Animal diseases pose significant challenges to livestock production and public health. This region faces various endemic and emerging diseases affecting cattle, sheep, goats and other animals. (VSF Germany 2020, Molla et al. 2010, Tekle et al. 2021, Sorsa 2022, Tschopp et al. 2010, Mebrahtu et al. 2018). The availability and quality of veterinary services are often limited due to factors such as resource constraints, environmental degradation and infrastructure gaps (Admassu 2003; Tesema 2017). Public veterinary services are reported to be inadequate in terms of both coverage and quality (Tesema 2017). These challenges are worsened by harsh climate conditions, limited access to modern veterinary services, traditional farming practices, poor management, a shortage of professionals and unrestricted movement of livestock. This has serious negative effects on the health and productivity of livestock, which are vital for the communities in the area.

In the Aari Zone, livestock play a central economic and cultural role, yet empirical evidence on community‐level experiences with veterinary services is scarce. It is believed that the absence of a culturally appropriate and sustainable approach to improving veterinary services in the zone perpetuates a cycle of inadequate animal healthcare and economic vulnerability for the rural communities. Therefore, engaging community members in identifying problems and collecting data is effective for identifying veterinary service gaps, especially when they centre local voices and foster collaboration.

Multiple studies demonstrate that the value of community‐engaged approaches in rural veterinary systems can significantly improve policy effectiveness by integrating local knowledge and enhancing community engagement in animal health interventions. The approach shifts the traditional top‐down service delivery models toward participatory research methodologies that integrate local knowledge with scientific expertise to improve policy outcomes. Participatory epidemiology enhances the involvement of animal keepers in analysing disease problems and designing control programmes (Catley et al. 2012). Another paper provides a concrete example, showing how dairy producers collaboratively developed antimicrobial stewardship policies that balanced health improvements with practical implementation (Van Dijk et al. 2017). Furthermore, CAHWs serve as critical information conduits in surveillance systems, particularly in remote pastoral areas where conventional veterinary services face significant constraints (Mariner et al. 2004). However, effective implementation requires careful attention to power dynamics and ensuring genuine community representation rather than superficial consultation (Ebata et al. 2020). The integration of transdisciplinary approaches that combine public health, socioeconomic, policy, veterinary and community perspectives has proven effective in developing comprehensive policy frameworks that address complex rural veterinary challenges (Chotinun et al. 2014; Namgyal et al. 2023).

Despite the importance of this study approach, there has been no research in the current study area. This study aims to address the gap in qualitative evidence by examining how livestock keepers in the Aari Zone experience and navigate barriers to veterinary services. It also aims to provide insights relevant to Ethiopia's wider agricultural extension landscape, where infrastructural and human resource challenges are shared across sectors. It was thus under such conditions that the problem was identified, providing a background to address the gap in veterinary services in the area. Therefore, this community‐engaged qualitative research was conducted to study current veterinary services and to identify key challenges in service delivery in the Aari Zone.

## 2 Materials and Methods

### 2.1 Description of the Study Area

**FIGURE 1:**
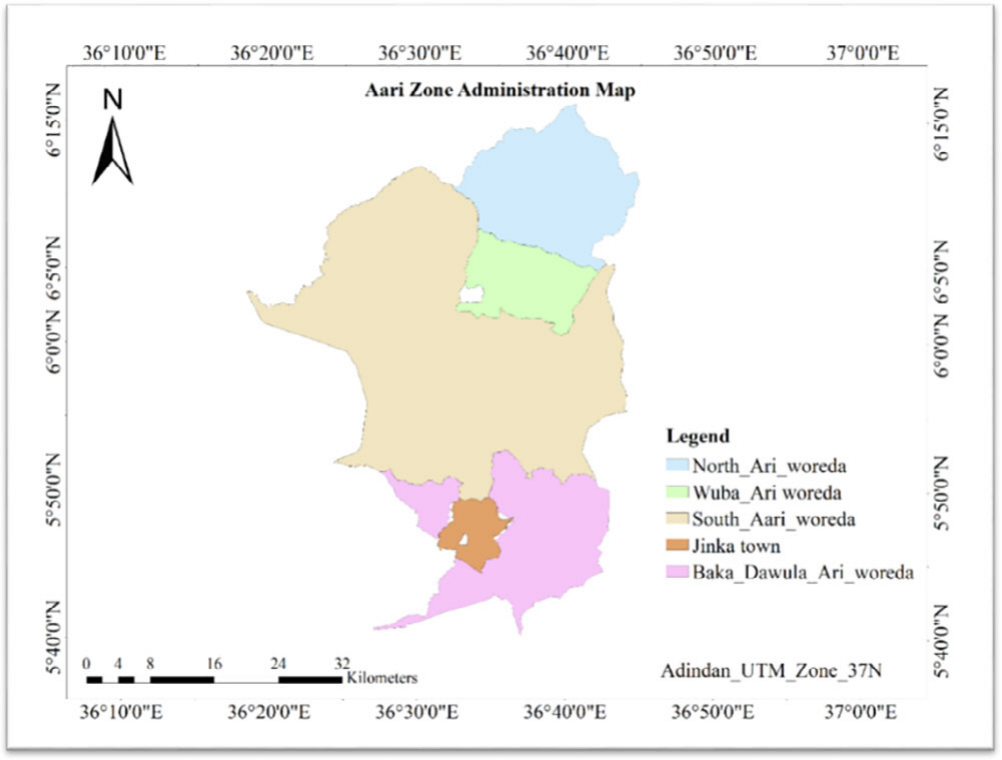
Map of the study areas (drawn with ArcMap).

The study was conducted in the Aari Zone of the Southern Ethiopia Region. Aari Zone is an administrative zone in the South Ethiopia Regional State. Until August 2023, Aari was part of the South Omo Zone. Jinka is the capital city of the zone. Jinka is characterized by diverse agro‐ecological conditions, an altitude of approximately 1390.17 masl, annual rainfall of 162.9 mm and average temperature of 22.07°C (Weather and Climate n.d.) Aari is bordered on the south by the south Omo Zone, on the northeast by the Gofa Zone and on the north by the Basketo Zone. The administrative centre of the Aari Zone is Jinka. Aari Zone comprises four districts (weredas) and two city administrations: Baka Dawla Aari, Debub Aari, Semen Aari and Woba Aari, as well as Gelila City Administration and Jinka City Administration (Figure 1). Aari Zone is characterized by diverse agro‐ecologies and predominantly agrarian rural communities, with mixed crop‐livestock systems. The Aari communities heavily rely on livestock raising, agriculture and natural resources for their livelihoods, cultural practices and food security. However, the region faces significant challenges in livestock health and productivity, impacting local livelihoods and food security. Veterinary infrastructure includes some kebele animal health posts (AHPs), farmer training centres (FTCs) and district agricultural offices, many of which face staffing and logistical challenges.

### 2.2 Study Methods

A community‐engaged, participatory‐informed qualitative research approach was employed using semi‑structured focus group discussions (FGDs) to explore both participants’ knowledge and lived experiences of veterinary service access, availability and quality. Community members had meaningful involvement in problem identification, data collection and validation; their input shaped the research questions. The community representatives helped identify veterinary service gaps as a priority problem, and later, while they did not co‐analyse data, findings were shared with community representatives for validation and feedback. This approach has been shown to generate practical, community‐driven solutions, such as improved information dissemination, culturally responsive service delivery, and targeted outreach (Weisent et al. 2024; Morales et al. 2025; Catley 2000), considering the community's technical level and culture, thereby increasing its acceptability.

### 2.3 Study Participants

Study participants were adult community members (livestock owners, traditional healers with animal health roles and community leaders) residing in the four districts. Before each FGD, we collected basic socio‐demographics (age range, gender, district/cluster, main livelihood and livestock ownership) via a brief pre‐discussion form administered orally by the facilitator and recorded in field notes.

### 2.4 Sampling Techniques

This study utilized a multistage cluster sampling method. The Southern Ethiopia Regional State was selected through purposive sampling, consisting of Jinka University (JKU), where the authors are based. Aari Zone was specifically selected as it is one of the catchment areas of JKU. Subsequently, all four districts within the zone, namely Baka Dawla Aari, Debub Aari, Semen Aari and Woba Aari, were acquired. A cluster was selected from Baka Dawla Aari and Woba Aari districts, as well as two clusters from Debub Aari and Semen Aari districts, based on the number of kebeles, with each cluster comprising 4–5 kebeles. Convenience sampling was used to select the participants of the FGD. The number of participants fell within the range of 5–11, which is recommended by scholars (Bernard 2017; Babbie 2020). A total of six FGDs (*n* = 54 participants) were conducted in the selected kebele clusters of the districts.

### 2.5 Data Collection

FGDs provide a platform for participants to explain and clarify their views and perspectives on commonly shared values, norms and knowledge (Krueger and Casey 2015; Howell 2013; Kitzinger 1995). The FGDs were conducted by the veterinarians (principal investigator and co‐investigators) with the support of the district animal health experts (two male and one female veterinarian). Prior to data collection, the research team held introductory meetings with relevant stakeholders, namely the Aari Zone and district agriculture office, kebele AHPs and kebele leaders to agree on study sites, community entry and suitable times/venues. The FGDs were conducted using in Amharic language, and the researchers probed further discussion arising from the FGDs. All discussions were translated into English following the discussions.

In the field, the FGDs were semi‐structured and facilitated by two veterinarians trained in qualitative facilitation. Discussions lasted 90–120 min, during which handwritten field notes were taken. Group dynamics were carefully managed to mitigate dominance and encourage equitable participation. To minimize power differentials between researchers and participants, we utilized locally recognized individuals to support recruitment and introductions, thereby avoiding coercion. FGDs were held in neutral, familiar venues (kebele community rooms/communal open spaces) at times selected by participants to reduce burden and perceived obligation. This helped research participants to freely communicate with the research team, and this created an atmosphere of openness. Where we detected imbalances (e.g., dominance by elders or local leaders), the facilitator re‑set ground rules, redistributed turns and explicitly invited quieter voices to contribute.

Primary data comprised facilitator field notes and post‑FGD debrief notes. We also conducted a document analysis of administrative documents and peer‑reviewed literature to contextualize findings.

### 2.6 Researcher Positionality

Our inquiry was guided by an interpretivist paradigm. Researchers were veterinarians and livestock development professionals with prior work experience in the region. Reflexivity was practised throughout, acknowledging potential power dynamics, professional background influence and the need for culturally respectful engagement.

### 2.7 Data Management and Analysis

A thematic approach (coding, theme development and triangulation) was used for data analysis (Ritchie and Lewis 2003). Analysis followed Reflexive Thematic Analysis (Braun and Clarke 2006), which is primarily inductive, progressing from open coding to candidate themes, such as familiarization with transcripts, generating initial inductive codes, collating codes into candidate themes, reviewing and refining themes, defining and naming themes and producing the final thematic structure. FGDs were translated, triangulated and analysed by the research team. Themes, sub‐themes and codes were produced, and visualizations (word cloud and word cloud bar chart) were generated using qualitative data analysis tools, such as ATLAS.ti 9.1.3.0 and Ailyze. Triangulation involved field notes, observations and secondary reports from district offices and relevant published scholarly articles.

### 2.8 Ethical Considerations

Ethical approval was obtained from the JKU Institutional Research Ethics Review Committee (Approval No. JKU/RERC/73/2017). Local permissions were obtained from the Aari Zone Agricultural Office and district authorities. Verbal informed consent was obtained from all participants after explaining the study aims, privacy protections and voluntary participation. Verbal consent for photography and publication of images was obtained separately. All data were stored on a password‐protected computer accessible only to the research team.

## 3 Results

### 3.1 Composition of FGD Participants

A total of 54 participants took part in six FGDs across four districts of the Aari Zone. Table 1 summarizes the distribution by cluster, gender and estimated age range. Participants were adults primarily engaged in livestock farming and related activities. Participants were adults, predominantly in their 30s and 40s, reflecting household heads and decision‐makers in livestock management. Gender composition varied by cluster, with overall representation of male (*n* = 38) and female (*n* = 16) participants. District representation was as follows: South Aari (33.3%), Bako Dawula (20.4%), North Aari (25.9%) and Wuba Aari (20.4%). This composition ensured diverse perspectives across geographic and socio‐cultural contexts within the zone.

**TABLE 1:** Information on research sites, gender and the number of FGD participants.

			Participant gender			
Districts/research sites	Number of FGDs	Age range (years)	Male	Female	Total participants	District representation (%)
South Aari	2					33.3
	Metser cluster	30–45	5	2	7	
	Gazer cluster	30–40	8	3	11	
Bako Dawula	1	30–47	10	1	11	20.4
North Aari	2					25.9
	Goza cluster	38–45	5	1	6	
	Safera cluster	38–43	6	2	8	
Wuba Aari	1	38–45	7	4	11	20.4
Total					54	100

### 3.2 Common Veterinary Service Gaps in the Areas

Analysis of the six FGDs (*n* = 54 participants) across four districts of Aari Zone revealed three major themes with associated sub‐themes. These themes capture common veterinary service gaps and contextual factors influencing service delivery. A summary of veterinary service gaps based on FGDs in the four districts of Aari Zone is presented in Table 2.

**TABLE 2:** Summary of veterinary service gaps and context in Aari Zone.

Theme	Sub‐themes	Key findings across districts
Theme I: Availability and Accessibility	1. Shortage and instability of professionals	Severe shortage of trained veterinarians; frequent absences for meetings/training; single staff covering multiple kebeles
	2. Long distances and transport challenges	Travel times up to 4 h; poor roads, especially during rainy season; remote kebeles underserved
	3. Inadequate clinics and AHPs	Uneven distribution of facilities; lack of cattle crushes for safe vaccination
	4. Limited‐service hours and interruptions	No night‐time/emergency services; weather disrupts campaigns
Theme II: Quality and Resources	1. Insufficient drug/vaccine supply	Irregular delivery; stockouts; expired drugs; reliance on costly private pharmacies
	2. Lack of diagnostics and scientific methods	No lab facilities; pregnancy diagnosis unavailable; reliance on syndromic treatment
	3. Need for training and monitoring	Low skill levels; demand for capacity building; gender‐specific challenges for female staff
	4. Inadequate infrastructure	Poor roads; lack of essential equipment (cattle crushes, PPE); limited clinic space
Theme III: Economic and Cultural Factors	1. Affordability issues	Costly private drug costs; lack of receipts; informal pricing
	2. Economic constraints	Feed shortages; poverty limits preventive care and timely treatment
	3. Limited cultural barriers	No cultural resistance to modern veterinary care; decisions are pragmatic
	4. Reliance on traditional practices	Traditional remedies used when modern services fail; some methods reduce market value

#### 3.2.1 Veterinary Service Availability and Accessibility

##### 3.2.1.1 Shortage and Instability of Veterinary Professionals

Participants consistently reported severe shortages and frequent absences of veterinary professionals, leading to service interruptions. Multiple FGDs confirmed frequent absences and unpredictable mobility of professionals, causing disruptions in kebele AHPs staffed by a single employee.

**FIGURE 2:**
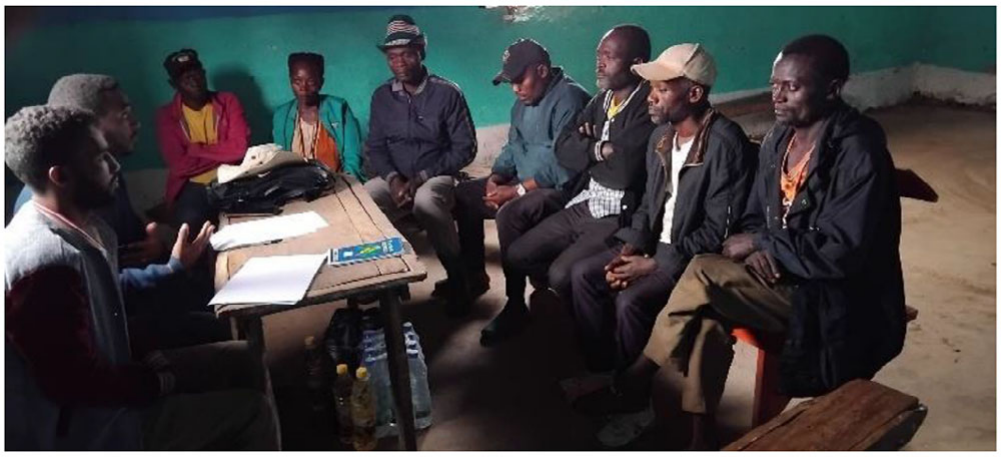
The FGD in the South Aari District.

A 34‐year‐old female participant in one of the FGDs in the South Aari district (Figure 2) highlighted the staggering workload:

*‘There is one professional for 21 development groups; because of the far distance, professionals can't arrive, and the animals may die’*. (South Aari, female, 34 years)

Professionals’ shortages or absences for meetings, training or emergencies cause critical service gaps, especially during vaccination campaigns. This limited staffing means areas without professionals receive no services. The 35‐year‐old FGD participant in Bako Dawula district and a participant in the FGD in South Aari district confirmed this service gap, stating:

*‘There is a shortage of professionals during the vaccination campaign; If the professionals are called away for meetings and training, the service will come to a halt, as we only have one employee available’*.

##### 3.2.1.2 Long Distances and Transportation Challenges

Long distances to veterinary facilities and poor transportation infrastructure critically hinder timely access to veterinary services in the present study area. Remote kebeles require long travel times (up to 4 h) to access services, which is worsened during rainy seasons.

*‘Accessing veterinary services often necessitates long‐distance travel; Some villages are quite remote, requiring animals to be transported for approximately four hours’*. (North Aari, male, 39 years)

The problem is compounded during rainy seasons when poor road conditions make travel difficult or impossible, as one of the key informants in the South Aari district said:

*‘Infrastructure (particularly, road, especially in the rainy season) has been our major problem for many years’*.

##### 3.2.1.3 Inadequate Veterinary Clinics and Animal Health Posts

The current study has also revealed that the physical infrastructure and equipment essential for veterinary services are widely inadequate, limiting service capacity and quality and becoming a critical constraint on service delivery. Several FGDs emphasized the inadequacy of cattle crushes and uneven distribution of kebele AHPs for safe animal handling and treatment.

*‘If there is no cattle crush, we can't get vaccination service’*. (South Aari, male participant)

**FIGURE 3:**
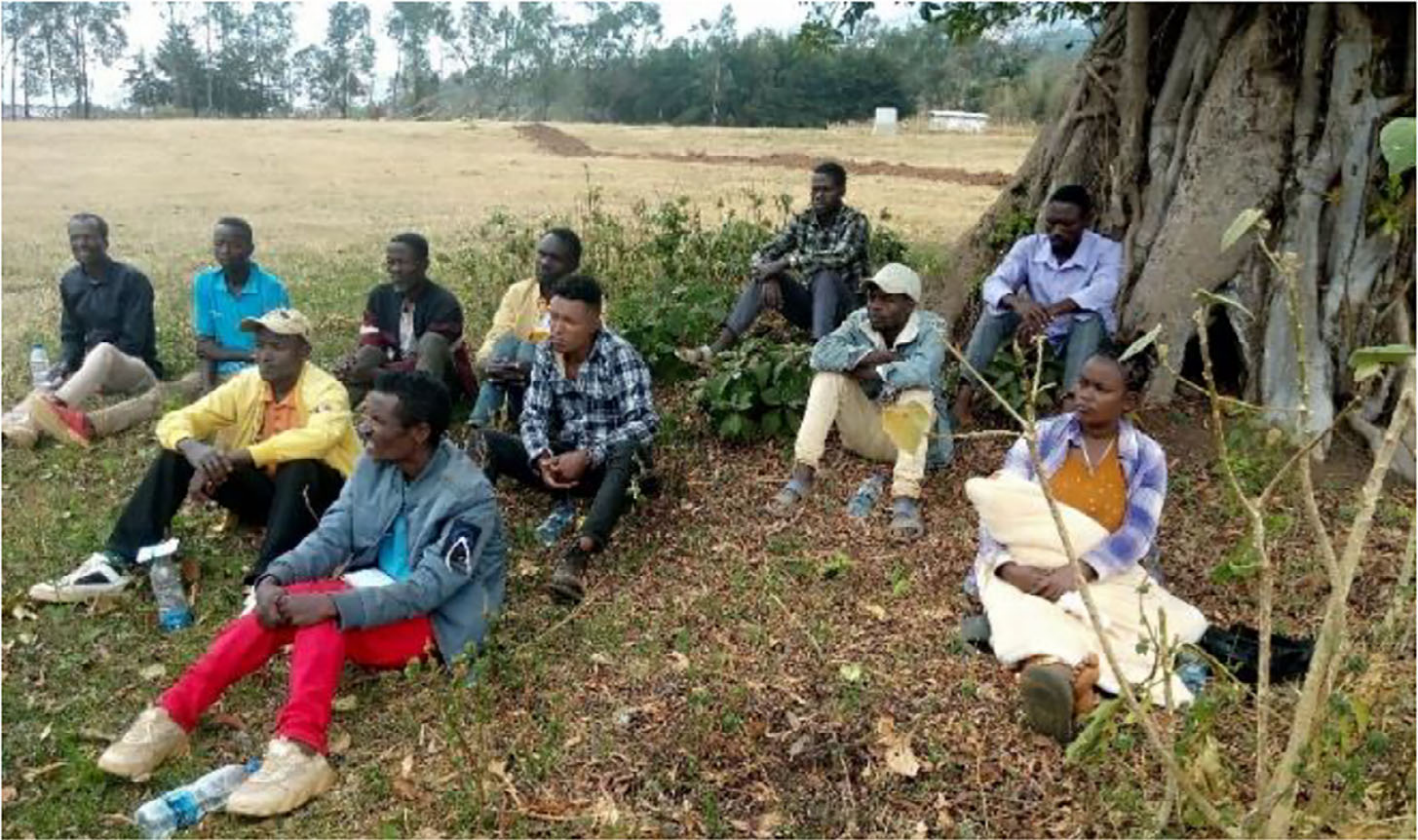
The FGD in the Wuba Aari District.

An FGD in South Aari district pointed to the ‘unfair distribution of clinics and cattle crushes’, indicating that many communities remain underserved. The problem is compounded by physical distance to facilities, as a Wuba Aari district FGD (Figure 3) reported, ‘There are kebele AHPs that are too far away from our home’, which restricts timely access. Professional shortages intensify these infrastructural deficits. Together, these findings reveal that both inadequate infrastructure and personnel shortages severely limit the functionality and accessibility of veterinary services in these areas.

##### 3.2.1.4 Limited‐Service Hours and Interruptions

The study showed that nighttime emergencies and adverse weather conditions disrupt service availability. Rainfall and seasonal weather significantly disrupt treatment provision and exacerbate travel difficulties, sometimes resulting in animal deaths. Notably, veterinary services are largely unavailable during nighttime, as the majority of the FGD participants in the South Aari district agreed:

*‘There is almost no veterinary service available during nighttime disease outbreaks, leading to critical gaps in emergency care. This lack of availability significantly hampers timely interventions and exacerbates the challenges faced by communities during health crises’*.

#### 3.2.2 Quality and Resources of Veterinary Services

##### 3.2.2.1 Insufficient Drug and Vaccine Supply

In the current study area, drug shortages and irregular vaccine delivery were common, forcing reliance on costly private pharmacies. The FGD in the Gazer cluster in the South Aari district confirmed the issue, stating:

*‘The supply of veterinary drugs and vaccines is insufficient and often not delivered on time, leading to delays in treatment and increased risks during disease outbreaks’*.

The irregular availability of drugs and vaccines forces farmers into costly private options. One farmer from Wuba Aari district elaborated on the issue, lamenting,

*‘We are currently wasting a significant amount of money at private pharmacies, often without receiving any service receipts. Our expenditures can reach as high as 1000 ETB, which raises concerns about transparency’*.

##### 3.2.2.2 Lack of Diagnostic Facilities and Scientific Methods

The communities in the current study area expressed a strong desire for evidence‐based, scientific treatment approaches. Absence of laboratory services and reliance on syndromic diagnosis limit treatment accuracy.

**FIGURE 4:**
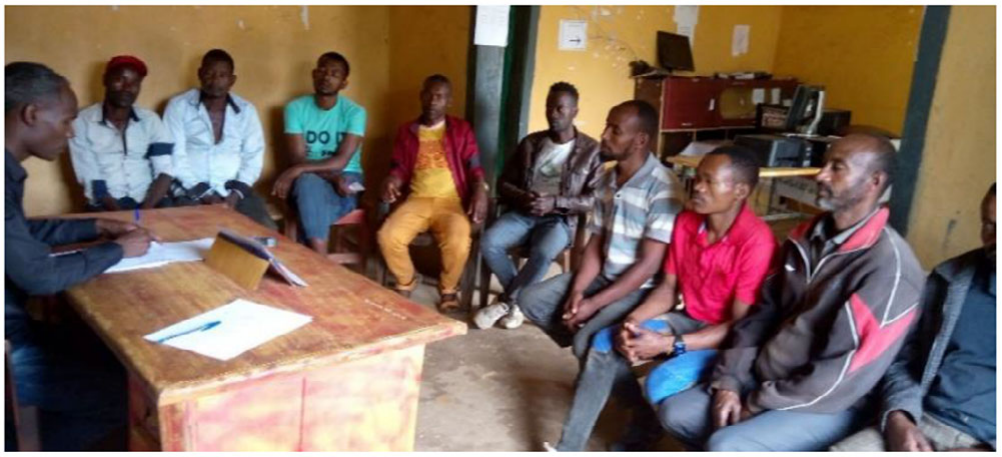
The FGD in the North Aari District.

Participants in the FGD from the Safera cluster in Semen Aari (Figure 4) and a model farmer from the South Aari district shared their thoughts on this issue, highlighting:

*‘We lack access to Pregnancy Diagnosis (PD) tests primarily due to a skills gap and insufficient materials, leading to an absence of accurate diagnoses during treatment. It is essential to enhance laboratory practices and ensure the availability of diagnostic materials’*.

##### 3.2.2.3 Need for Professional Training and Monitoring

Communities emphasized low skill levels among professionals and requested capacity‐building initiatives. There is broad recognition of the need to enhance veterinary professionals’ skills through training and capacity building. FGDs in Semen Aari district underscore this with calls for ‘training the animal health expert and capacity building’,

*‘The skill of professionals is low; training is needed’*. (Wuba Aari FGD)

Special attention is needed to support female veterinary staff, as one of the participants from the FGD in the South Aari district revealed that ‘*female professionals often experience fear and a lack of confidence’*, indicating gender‐specific challenges.

Multiple FGDs have also revealed that professionals are often absent during critical periods and reside far from their service sites, which also results in service gaps. One FGD participant in the Bako Dawula district stated:

*‘In places where professionals are not present, there is a complete lack of services. Their absence severely limited access to essential resources for the local community’*.

##### 3.2.2.4 Inadequate External Infrastructure

Poor roads, in addition to the lack of essential veterinary equipment, hinder service delivery, as stated by several FGDs.

*‘Infrastructure, especially roads during the rainy season, has been our major problem for many years’*. (South Aari FGD)

#### 3.2.3 Economic and Cultural Factors Affecting Veterinary Care

##### 3.2.3.1 Affordability Issues, Especially Private Pharmacy Services and Drug Costs

**FIGURE 5:**
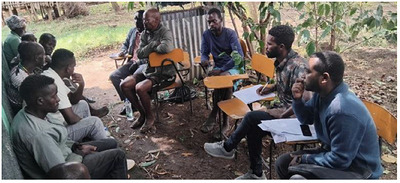
The FGD in the Bako Dawula District.

The study also showed that the communities have noted that high costs at private pharmacies and informal charges create financial strain. The limited availability of drugs in public facilities forces dependence on more expensive private sellers. Participants in the FGDs in Bako Dawula district (Figure 5 ) have addressed this concern, reflecting:

*‘The costs associated with purchasing treatment from a private pharmacy are becoming unaffordable, with prices reaching as high as 300 ETB. This financial burden poses a significant challenge for us, making it difficult to sustain necessary healthcare services’*.

*‘We are wasting a significant amount of money at private pharmacies, often without receipts’*. (Wuba Aari FGD)

Illegal drug marketing and poor‐quality control exacerbate these problems, with FGD in Semen Aari district noting issues such as expired drugs and unskilled sellers.

##### 3.2.3.2 Economic Constraints

The present study revealed that economic barriers critically restrict livestock owners’ ability to access veterinary services, with high drug prices and unaffordable private pharmacy costs at the forefront. These economic hardships directly contribute to inadequate treatment and livestock mortality, threatening farmers’ livelihoods and productivity.

Economic factors beyond drug costs, such as forage shortages and poverty, further impair livestock health, nutrition and productivity. Participants in the FGD from Metser, South Aari district, expressed their frustrations regarding the lack of forage, stating:

*‘We are facing a shortage of forage in both supply and the area available for planting, which indicates a critical limitation in our feed resources. This scarcity not only affects the nutritional needs of our livestock but also reduces overall productivity’*.

##### 3.2.3.3 Limited Influence of Cultural Beliefs on Modern Veterinary Service Adoption

FGDs in the current study showed that, cultural beliefs do not present barriers to the adoption of modern veterinary services, indicating a supportive cultural environment. All FGDs in the four districts consistently state that there are ‘no cultural beliefs that influence modern treatment’ and ‘no traditional beliefs that challenge the adoption of modern treatment technologies’. The decisions to seek veterinary care are driven by practical considerations such as service quality and availability rather than cultural resistance.

##### 3.2.3.4 Continued Reliance on Traditional Practices

The FGDs in different areas of the Aari Zone revealed that traditional veterinary practices play an important complementary role, especially where modern veterinary services are inaccessible, ineffective, or unsatisfactory. An FGD in Bako Dawula district emphasized the usage of traditional practices ‘in places where they are far away from the centre’. Furthermore, traditional treatments are crucial for diseases ‘not able to be treated with modern treatment’, as noted by FGD participants in Gazer, South Aari district, and remain relevant for severe cases or in remote areas, as indicated in the FGD in Metser, South Aari district. Despite a community shift towards modern methods, traditional practices persist as essential fallback options, contributing to comprehensive livestock health management.

*‘We prefer traditional means to cure our animals when experts are not available’*. (Bako Dawula FGD)

A consolidated table of findings by theme and district is presented below (Table 2).

## 4 Discussions

Six FGDs (*n* = 54 participants) across four districts of Aari Zone were conducted and revealed three major themes with associated sub‐themes.

The demographic composition of the focus group participants in Table 1 has important implications for interpreting the findings and designing future interventions. The age range of 30–47 years across clusters indicates that participants were primarily household heads and decision‐makers in livestock management. This suggests that the insights gathered reflect the perspectives of individuals with significant responsibility for animal health and resource allocation, which strengthens the relevance of the recommendations for improving veterinary service delivery. However, the limited representation of younger adults and the relatively lower proportion of women (16 out of 54 participants) highlight potential gaps in capturing the experiences of groups who may face distinct barriers.

Barriers to veterinary service access in Ethiopia are multifaceted. The most significant barriers include poor infrastructure, financial constraints, shortages of skilled personnel and limited availability of drugs and equipment. The consequences of these workforce challenges are significant.

In the present study, the shortage, absence and instability of veterinary professionals are affect the animal health sector, undermining the effective delivery of veterinary services. Community members in the present study explicitly recognize these shortages, emphasizing the need for ‘Employment of an adequate number of professionals’. Communities consistently advocate for the employment of stable, skilled personnel and support for existing workers to ensure continuous, reliable veterinary services, emphasizing workforce stability as fundamental to service availability. Several studies in Ethiopia report a critical shortage of trained veterinary professionals, especially in rural and pastoral areas. A pilot survey in Hawassa, Shashemene and Negele Arsi found that a clear shortage of qualified personnel was cited as a limiting factor for effective service delivery (Tafa et al. 2023). Similarly, a review of pastoral areas emphasized that the shortage of trained manpower is a persistent constraint, directly impacting livestock productivity and disease control (Jilo 2016). Studies in central Ethiopia and the Sidama Region found that government clinics often operate with insufficient staff, leading to high dissatisfaction among both professionals and livestock owners (G. Kebede et al. 2025; Tessema and Negash 2022; Mulugeta et al. 2019).

The current study has also revealed that long distances to veterinary facilities and poor transportation infrastructure are becoming a barriers to veterinary service access. These communities’ insights demonstrate that geographic and infrastructural barriers are central determinants of veterinary service accessibility and timely veterinary care, negatively impacting animal health. Infrastructure and geographic barriers limit the establishment and accessibility of veterinary clinics and services. Several studies support this concern, describing that distance to service centres is a major factor; livestock owners in remote areas often travel long distances, discouraging timely care (Gizaw et al. 2021; Desta 2015; H. Kebede et al. 2014; Jilo 2016).

The study findings in Aari Zone have also shown that infrastructure and equipment essential for veterinary services are widely inadequate and are becoming a critical constraint on service delivery. This is in agreement with many studies (Tafa et al. 2023; Gizaw et al. 2021; Desta 2015; Tessema and Negash 2022; Nyokabi et al. 2024; H. Kebede et al. 2014; Fedlu and Seid 2019; Jilo 2016), which describe how many facilities lack basic equipment, diagnostic tools and proper storage for medicines, making timely and effective service delivery difficult. Most clinical cases are diagnosed based only on clinical signs due to a lack of laboratory support and skilled diagnosticians (Nyokabi et al. 2024; Tessema and Negash 2022; Etefa et al. 2021). This leads to irrational drug use, poor disease surveillance and limited capacity to respond to outbreaks.

FGDs in this study have also revealed that frequent interruptions in veterinary services have increased due to adverse climatic factors and the lack of night‐time veterinary services. As noted by the present communities, these factors limit continuous service availability and negatively influence animal health outcomes, further illustrating service gaps. These combined human and environmental factors disrupt continuous and accessible veterinary care, with severe consequences for livestock health.

Adverse weather directly disrupts veterinary service delivery. These conditions can limit the mobility of veterinary professionals, making it difficult to reach remote or pastoral communities, especially during emergencies (Stephen and Soos 2021; Gizaw et al. 2021). They increase the incidence and spread of animal diseases, raising demand for services at times when access is most difficult. Furthermore, they exacerbate resource shortages, as climate events can disrupt supply chains for medicines and equipment (Akello 2024; Stephen and Soos 2021), while also damaging infrastructure such as roads and clinics, further reducing service availability and reliability (Stephen and Soos 2021; Gizaw et al. 2021). The absence of night‐time or emergency veterinary services is a critical gap. One study has shown that livestock keepers report low satisfaction with the timeliness and availability of services, particularly during nights and weekends when emergencies often occur. This gap leads to increased animal losses and economic hardship, as urgent cases may go untreated until regular hours. The lack of 24‐h service is more pronounced in rural and pastoral areas, where alternative options are limited (Gizaw et al. 2021).

KIs in the present study areas have expressed their concern regarding inadequate and irregular drug and vaccine supplies. They have also raised economic constraints related to the high drug prices and unaffordable private pharmacy costs, illegal drug marketing and poor quality control. Such supply chain weaknesses compromise both preventive and curative veterinary interventions, reducing livestock protection and health outcomes. Different studies have showed that public veterinary clinics frequently experience drug shortages, with many public providers reporting this as a major issue (Tafa et al. 2023; Tessema and Negash 2022; Mekonen et al. 2024; G. Kebede et al. 2025; Gizaw et al. 2021; Asfaw et al. 2021). Robi et al. (2024) and Asfaw et al. (2021) confirmed the impact of low vaccine supply, stating that vaccine availability is limited and distribution is irregular, leading to low vaccination rates and poor disease control. High costs of private veterinary services and drugs further limit access, especially for poorer farmers and pastoralists, as noted by Tafa et al. (2023), Gizaw et al. (2021), Mulugeta et al. (2019), G. Kebede et al. (2025) and Pasteur et al. (2024). Both public and private sectors are affected: public services are hampered by manpower shortages and resource constraints, while private services face challenges such as black‐market drug dealers and high service costs (Jibat et al. 2015; G. Kebede et al. 2025; Mulugeta et al. 2019).

Illegal drug marketing and the presence of substandard or counterfeit drugs are widespread, especially in remote areas, undermining treatment effectiveness and contributing to antimicrobial resistance (Desta 2015; Gizaw et al. 2021; Etefa et al. 2021; H. Kebede et al. 2014). Weak regulation of private providers, as well as a lack of integration between public and private sectors, further complicates service delivery, allowing the circulation of ineffective or expired drugs (Mekonen et al. 2024, Gizaw et al. 2021; H. Kebede et al. 2014; Desta 2015).

Livestock keepers in the four districts of Aari Zone have well‐recognized that the shortage of training and capacity building among veterinary professionals is a barrier to effective veterinary service access. However, training efforts face operational difficulties due to limited staffing, highlighting the risk of service interruptions during capacity‐building activities. These findings suggest that training programmes must be carefully planned alongside workforce expansion to ensure continuous service delivery and empowerment of veterinary professionals. Other studies have also shown that veterinary staff at district and community levels often lack in‐depth training in disease surveillance, outbreak investigation and One Health approaches. This results in lower experience and effectiveness in animal health service delivery, particularly in rural and underserved areas (Auplish et al. 2024; Subharat et al. 2023). Surveys of Ethiopian veterinary professionals reveal a strong demand for training in laboratory diagnostics, disease prevention, antimicrobial resistance, epidemiology and clinical procedures. Many professionals feel underprepared in these areas, which limits their ability to deliver high‐quality services (Alafiatayo et al. 2022; Bessler et al. 2024; Nyokabi et al. 2024). Similar challenges are reported in Kenya, Vietnam, India and other countries, where capacity‐building programmes and targeted training have been shown to improve workforce competence and service delivery (Sitawa et al. 2023; Rao et al. 2015; Kinnison et al. 2020). The lack of regular training and capacity building leads to over‐reliance on syndromic knowledge and experience rather than evidence‐based practices. This reduces diagnostic accuracy, disease surveillance and outbreak response capacity (Nyokabi et al. 2024; Tessema and Negash 2022; G. Kebede et al. 2025; Gizaw et al. 2021). Professionals with less training are less likely to adopt best practices, participate in multisectoral collaboration, or respond effectively to disease outbreaks. This directly limits the reach and quality of veterinary services (Auplish et al. 2024; Sitawa et al. 2023, Auplish et al. 2024; Subharat et al. 2023).

The FGDs in different areas of the Aari Zone revealed that traditional veterinary practices play an important complementary role, especially where modern veterinary services are inaccessible, ineffective or unsatisfactory. This coexistence underscores the importance of strengthening modern veterinary services to reduce reliance on traditional methods where possible. Traditional veterinary practices are vital in bridging service gaps in Ethiopia and globally. Ethnoveterinary practices are recognized worldwide, with research highlighting their practical applications in disease control, diagnostics and herd management. In places like China and southern Europe, traditional herbal medicines and remedies are being studied for integration into evidence‐based veterinary care (Schillhorn Van Veen 1997; Xu et al. 2024; Pieroni et al. 2004). Ethiopia's diverse ethnic groups maintain a rich array of traditional veterinary knowledge. These methods are especially important in rural and pastoralist communities where modern services are scarce or unaffordable (Mesfin and Obsa 1994). Traditional practices empower local farmers and herders to address animal health issues cost‐effectively, using locally available resources. This is particularly valuable in areas with limited infrastructure or during drug/vaccine shortages (Schillhorn Van Veen 1997; Mesfin and Obsa 1994).

The veterinary service gaps identified in Aari Zone mirror structural challenges across Ethiopia's wider agricultural extension architecture. Ethiopia's extension system operates through FTCs and Development Agents (DAs) under the Ministry of Agriculture, designed to deliver crop, livestock and natural resource management services at kebele level. However, similar to the Aari context, these centres often face human resource shortages, infrastructure deficits and logistical constraints that undermine service quality and reach (Alafiatayo et al. 2022; Gizaw et al. 2021; Nyokabi et al. 2024; Wonde et al. 2022; Habane et al. 2025; A. Alemu 2021). Addressing these gaps requires multi‐level, integrated policy responses aligned with Ethiopia's rural development, such as strengthening public‐private partnerships, investing in infrastructure, continuous professional development and One Health strategies (Bessler et al. 2024; Nyokabi et al. 2023; Braam et al. 2023; Nyokabi et al. 2024; Alafiatayo et al. 2022).

### 4.1 Limitations of the Study and Future Directions

This study incorporated community engagement but did not fully meet the principles of Community‐Based Participatory Research (CBPR) co‐design and co‐analysis. While FGDs were valuable for exploring community norms and shared experiences, they may have underrepresented sensitive or private issues. Although efforts were made to minimize bias, power dynamics between researchers and participants may have influenced the discussions. The findings are specific to the Aari Zone and should therefore be interpreted within this context, although they may offer transferable insights for similar rural settings. In addition, translation of data from Amharic into English may have resulted in some loss of linguistic and cultural nuance; however, back‑checking was performed on all quoted passages. The selected districts may not fully reflect the experiences of more remote or highly mobile households. Future research should focus on documenting, evaluating and integrating safe and effective ethnoveterinary practices into formal animal health systems, while policymakers and stakeholders should strengthen public‐private partnerships, invest in rural infrastructure and support continuous professional development for animal and human health professionals to improve service accessibility, quality and sustainability.

## 5 Conclusions

The collective analyses revealed that the veterinary service delivery in the studied regions is hampered by intertwined challenges spanning workforce shortages and instability, infrastructural inadequacies, geographic and transportation barriers, limited service hours, supply chain deficiencies, skill gaps and economic constraints. Addressing these issues demands comprehensive strategies that enhance professional recruitment and training, expand and equitably distribute veterinary infrastructure, improve drug and vaccine supply chains, develop diagnostic and laboratory capacities, ensure affordability and transparency in service payments and adapt service delivery models to local geographic and climatic realities. These holistic approaches are crucial to overcoming the multifaceted challenges confronting veterinary service delivery and, hence, are essential to improving veterinary service availability, accessibility, quality and ultimately, livestock health and community livelihoods in the Aari Zone. Moreover, acknowledging and integrating traditional veterinary practices where appropriate can enhance overall livestock health management. Addressing these interconnected issues holistically will be essential to strengthening veterinary services and supporting the health and productivity of livestock‐dependent communities.

## Author Contributions

**Yebelayhun Mulugeta**: conceptualization, data curation, formal analysis, writing – original draft, writing – review and editing. **Taye Shibabaw**: data curation, writing – review and editing. **Haile Delelegn**: data curation, writing – review and editing. **Belete Jorga**: data curation, writing – review and editing. All authors approved the final version of the manuscript to be published and agreed on the journal to which the article has been submitted.

## Funding

This work was funded by Jinka University, Jinka, Ethiopia (Grant Number: GOV/JKU/TH7/CANR/DVM/2017).

## Ethics Statement

Permission letter (ethical clearance) to undertake the research was granted by the Jinka University Institutional Research Ethics Review Committee (Approval No: JKU/RERC/73/2017). Before conducting the study, verbal informed consent was obtained from the livestock owners included in this study. Participation of all respondents in the survey was strictly voluntary. There was no harm to the study participants. Measures were taken to ensure the respect, dignity and freedom of each participating individual in the interview. Appropriate measures were taken to ensure the confidentiality of the information both during and after data collection. Permission has been given by the individuals to use their images in this manuscript. All data were stored on a password‐protected computer accessible only to the research team.

## Conflicts of Interest

The authors declare no conflicts of interest.

## Data Availability

The data used to support the findings of this study are available at Jinka University, Jinka, Ethiopia.
